# Situation and cognition on the application of mechanical ventilation: a baseline investigation in northwest china

**DOI:** 10.1186/2197-425X-3-S1-A147

**Published:** 2015-10-01

**Authors:** L Song, Y Wu, J Chen, Z Li

**Affiliations:** Xijing Hospital, Forth Military Medical University, Pulmonary and Critical Care Medicine, Xi'an, China

## Introduction

With the progress of medical technology especially mechanical ventilation (MV), more and more hospitals have been buying ventilators in China. But the system of respiratory care has been not set up in this country. The situation of MV is worth to be is worth paying attention to.

## Objectives

The aim of this study was to investigate the present situation and cognition level on the application of MV in the above second-class hospitals in Northwest China, where there was almost no respiratory therapist.

## Methods

All 192 trainees who came from Northwest China were enrolled in a ventilator-training class and would finish the questionnaires.

## Results

181 qualified questionnaires were collected. The respondents came from upper first-class hospitals (45.5%), middle first-class (11.3%), upper second-class (39.5%) and middle second-class (3.7%). In 75.2% hospitals doctors were responsible for operating ventilators, in 1.2% nurses and in 24.6% both of them did it. On the actual results of MV, 80.2% respondents thought using MV in early time could improve the patients´ condition. But 14.8% thought the main aim of MV was satisfying the request of family, 38.2% thought MV only could retard the disease in short-term without long-term meaning, 20.9% thought MV was expensive with poor result and 4.9% thought MV would double the pain of patients. Many complications in using non-invasive ventilation (NIV) could be meet, such as expectoration difficulties (70.3%), abdominal distension (58%), aspiration (25.9%), facial pressure ulcers (24.7%), unstable blood pressure (8.6%), pneumothorax (12.3%). 8.6% respondents routinely gave sedation to NIV patients, 11.1% did it for most of time, 25.9% occasionally and 8.3% never. There were a lot of confusion in the NIV application, such as not knowing clearly the structure and mechanisms of ventilator (61.7%), not grasping exactly the indications of NIV (67.9%), not being sure of adjustment methods (75.3%) and not knowing how to resolve the carbon dioxide retention (53.1%).

## Conclusions

In the most of Northwest China hospitals doctors played main role in operating ventilators. Because ventilators were distributed in different departments and some operators were lack of systematically training, incorrectly use caused rather more complications and lower success rate, and reduced the confidence of staff and patient family in MV application. So it is necessary for China to set up a sort of rational system of respiratory care.

